# Predicting 30‐day mortality in older patients with suspected infections by adding performance status to quick sequential organ failure assessment

**DOI:** 10.1002/jgf2.764

**Published:** 2025-02-05

**Authors:** Masataka Kudo, Sho Sasaki, Toshihiko Takada, Kotaro Fujii, Yu Yagi, Tetsuhiro Yano, Ken‐ei Sada, Shunichi Fukuhara, Narufumi Suganuma

**Affiliations:** ^1^ Department of General Internal Medicine Iizuka Hospital Fukuoka Japan; ^2^ Department of Clinical Epidemiology Kochi Medical School Nankoku Japan; ^3^ Department of Internal Medicine Inan Hospital Kochi Japan; ^4^ Section of Education for Clinical Research Kyoto University Hospital Kyoto Japan; ^5^ Clinical Research Support Office Iizuka Hospital Fukuoka Japan; ^6^ Department of General Medicine, Shirakawa Satellite for Teaching and Research (STAR) Fukushima Medical University Fukushima Japan; ^7^ Department of Healthcare Epidemiology, School of Public Health in the Graduate School of Medicine Kyoto University Kyoto Japan; ^8^ Academic and Research Centre Hokkaido Centre for Family Medicine Sapporo Japan; ^9^ Section of Clinical Epidemiology, Department of Community Medicine, Graduate School of Medicine Kyoto University Kyoto Japan; ^10^ Medical School, Medical Course, Department of Human Health and Medical Sciences Kochi Medical School Nankoku Japan

**Keywords:** activities of daily living, aged, organ dysfunction scores

## Abstract

**Background:**

Quick Sequential Organ Failure Assessment (qSOFA) is a simple and easy tool for identifying patients with suspected infection, who are at a high risk of poor outcome. However, its predictive performance is still insufficient. The Eastern Cooperative Oncology Group Performance Status (ECOG‐PS) score, a tool to evaluate physical function, has been recently reported to be useful in predicting the prognosis of patients with pneumonia. We aimed to evaluate the added value of ECOG‐PS to qSOFA in predicting 30‐day mortality in older patients admitted with suspected infections.

**Methods:**

Between 2018 and 2019, we prospectively collected data from adults aged 65 years or older, admitted with suspected infection at two acute care hospitals. Predictive performance was compared between two logistic regression models: one using qSOFA score alone (qSOFA model) and the other in which ECOG‐PS was added to qSOFA (extended model).

**Results:**

Of the 1536 enrolled patients, 135 (8.8%) died within 30 days. The area under the curve of the extended model was significantly higher than that of the qSOFA model (0.67 vs. 0.64, *p* = 0.008). When the risk groups were categorized as follows: low (<5%), intermediate (5%–10%), and high (≥10%), 5.0% of those who died and 2.1% of those who survived were correctly reclassified by the extended model with an overall categorized net reclassification improvement of 0.03 (95% confidence interval: −0.06 to 0.30).

**Conclusions:**

Adding the ECOG‐PS score could improve the performance of qSOFA in predicting mortality in older patients admitted with suspected infection.

## Abstract 2

The extended model (ECOG‐PS + qSOFA) significantly improved discrimination in predicting mortality among older patients with suspected infections compared to the model with qSOFA alone (*p* = 0.008), with the net reclassification index revealing that 21 (17.5%) of the deceased were elevated to a higher risk category. This approach integrates routinely measured functional measures for enhanced risk assessment.

## 1 BACKGROUND

The mortality rate of sepsis is high, reaching up to 19% in older patients.^1^, ^2^ The identification of patients at high risk of mortality is crucial, as the early initiation of antibiotics and care bundles improve prognosis.^3^ The Quick Sequential Organ Failure Assessment (qSOFA) score, which measures three vital signs, is a simple tool that predicts mortality in patients outside the intensive care unit (ICU).^1^ The median area under the receiver operating characteristic (AUROC) for in‐hospital mortality was 0.68, with an interquartile range (IQR) of 0.55–0.82. The median (IQR) sensitivity and specificity for in‐hospital mortality were 52% (16%–98%) and 81% (19%–97%), respectively.^4^ This score shows a high degree of specificity in predicting mortality; however, its low sensitivity means that it may not predict mortality, particularly in older patients. In a study evaluating the mortality‐predicting ability of qSOFA in patients aged 65 years or older, admitted to an Intermediate Care Unit with an in‐hospital mortality rate of 36.9%, the sensitivity and specificity of qSOFA were 0.53 and 0.75, respectively. Older patients often have multiple baseline comorbidities, which may influence the assessment of qSOFA scores and should not be determined by qSOFA alone.^5^ To improve the performance of qSOFA without decreasing its simplicity, combining it with a predictor that is easily available in daily clinical practice would be useful.

Physical activity is associated with the prognosis of older patients with acute illnesses.^6^ The Eastern Cooperative Oncology Group‐Performance Status (ECOG‐PS) is a simple tool that evaluates a patient's daily physical functions on a 6‐point scale; it offers sufficient reliability compared with other PS measurement tools.^7^ Although ECOG‐PS was initially developed for patients with cancer,^8^ it has recently been widely used in other patients. For instance, the ECOG‐PS score has been associated with mortality in patients who underwent emergency abdominal surgery^9^ and those with community‐acquired pneumonia (CAP).^6^ Among patients with CAP, the addition of ECOG‐PS to confusion, uraemia, respiratory rate, blood pressure (BP), age ≥65 years, all of which together comprise the CURB‐65 score, which is a tool for predicting the severity of CAP, significantly improved the sensitivity of mortality prediction.^6^ To date, no study has analyzed the value of adding the ECOG‐PS score to the qSOFA score for predicting mortality in older patients with suspected infections.

In this study, we evaluated the value of adding the ECOG‐PS score to the qSOFA score for predicting mortality in older patients admitted with suspected infectious diseases.

## 2 METHODS

### 2.1 Study design and setting

This prospective observational study was conducted in the Department of General Medicine of two acute care teaching hospitals: Iizuka Hospital (1048‐bed capacity, Fukuoka, Japan) and Shirakawa Kosei General Hospital (471‐bed capacity, Fukushima, Japan). This study was conducted in accordance with the Declaration of Helsinki and the Ethical Guidelines for Epidemiological Research in Japan. The ethics committees of both hospitals approved this study (approval number: 17135 for Iizuka Hospital and HAKURIN17‐003 for Shirakawa Kosei General Hospital). In compliance with the Ethical Guidelines for Medical and Health Research Involving Human Subjects in Japan, written consent was obtained at Shirakawa Kosei General Hospital per the ethics committee's instructions, while an opt‐out consent method was used at Iizuka Hospital. The Standards for Reporting of Diagnostic Accuracy Studies (STARD) guidelines were followed to ensure transparency in reporting.^10^

### 2.2 Selection of participants

Between January 2018 and January 2019, we consecutively enrolled patients aged ≥65 years admitted with suspected infections. Patients with suspected infections were defined as those who underwent at least two sets of blood culture examinations within 24 h of admission to the Department of General Medicine.^11^, ^12^, ^13^, ^14^ Patients' medical care providers decided to obtain blood samples based on their discretion.

### 2.3 Measurements

Using a structured collection form, we collected data from the electronic medical records of each hospital.

#### 2.3.1 Quick sequential organ failure assessment (qSOFA)

The qSOFA score ranges from 0 to 3, and 1 point is assigned for each of the following conditions: hypotension (systolic blood pressure ≤100 mm Hg), tachypnoea (respiratory rate ≥22/min), and altered mentation (Glasgow Coma Scale [GCS] score <15).^1^ In older patients, a GCS score of <15 may occur without any change from their usual state. Therefore, we also confirmed any deviations from their usual state with family members or caregivers.

#### 2.3.2 Eastern Cooperative Oncology Group‐Performance Status (ECOG‐PS)

The ECOG‐PS scores ranged from 0 to 5.^8^ Grade 0 of ECOG‐PS is defined as “fully active, and able to carry on all pre‐disease performances without restriction”; Grade 1, “restricted in physically strenuous activity but ambulatory and able to carry out work of a light or sedentary nature, for example, light house work and office work”; Grade 2, “ambulatory and capable of all selfcare but unable to carry out any work activities; up and about for >50% of waking hours”; Grade 3, “capable of only limited selfcare; confined to bed or chair for >50% of waking hours”; and Grade 4 as “completely disabled; cannot carry out any self‐care; totally confined to a bed or chair.” Because Grade 5 was defined as death, patients with ECOG‐PS scores of Grade 5 were not included (Table 1). The Japanese version of the ECOG‐PS questionnaire was obtained from the Japan Clinical Oncology Group website.^15^ The attending physicians assigned ECOG‐PS scores based on interviews with the patients or caregivers during admission.

**TABLE 1:** Definition of ECOG‐PS grades.

ECOG‐PS grade	Description
0	Fully active, able to carry on all predisease performances without restriction
1	Restricted in physically strenuous activity but ambulatory and able to carry out light or sedentary work (e.g., light housework, office work)
2	Ambulatory and capable of all self‐care but unable to carry out any work activities; up and about for >50% of waking hours
3	Capable of only limited self‐care; confined to bed or chair for >50% of waking hours
4	Completely disabled, cannot carry out any self‐care, totally confined to a bed or chair
5	Death (not included in the study)

Abbreviation: ECOG‐PS, Eastern Cooperative Oncology Group Performance Status.

### 2.4 Outcomes

The primary outcome measure was mortality within 30 days of admission, which was confirmed by reviewing the electronic medical records. Patients whose survival status at 30 days could not be confirmed owing to transfer or discharge from the hospital were defined as surviving.

### 2.5 Other variables

On admission, the following variables were evaluated by reviewing the electronic medical records: age, sex, body mass index (BMI), body temperature, diastolic blood pressure, heart rate, comorbidities, status of immunosuppressive treatment, laboratory data (white blood cell count, platelet count, C‐Reactive Protein (CRP) level, serum albumin level, and serum creatinine level), and whether the patient's final diagnosis was a bacterial infection.

### 2.6 Statistical analyses

Data are expressed as frequencies and percentages for categorical variables and as medians and IQR for continuous variables.

First, baseline characteristics of surviving and deceased patients were compared using chi‐squared analysis for categorical variables and Student *t*‐test for continuous variables.

Second, sensitivity (Sn), specificity (Sp), positive and negative predictive values (PPVs and NPVs), and positive and negative likelihood ratios (LR+ and LR−) of qSOFA and ECOG‐PS scores for 30‐day mortality were calculated. Scores of 2 and 3 for qSOFA and ECOG‐PS, respectively, were used as cutoff points.^1^

Subsequently, we compared the predictive performances of two logistic regression models to estimate the risk of mortality as follows: in the first model, we used only the qSOFA score (qSOFA model); in the second model, we added the ECOG‐PS score to the qSOFA model (extended model). We constructed a receiver operating characteristic (ROC) curve and assessed its discriminative performance by analyzing the area under the curve (AUC). Calibration of the model was visually assessed using a calibration plot.^16^ We evaluated the degree of overfitting in terms of the optimism‐corrected AUC, calibration slope, and calibration‐in‐the‐large by performing internal validation using bootstrap techniques.^17^

Finally, we calculated the net reclassification improvement (NRI) value to assess the improvement in predictive performance for 30‐day mortality upon the addition of the ECOG‐PS score to the qSOFA score.^18^ Using the categorized NRI values, we separately calculated the proportion of deceased and surviving patients who moved up or down the risk strata. The overall NRI value was the sum of the improvements in each set.^19^ We used a reclassification table to visualize the number of patients who moved to another or remained in the same risk category.^19^ The participants were divided into three risk groups: low risk (<5%), intermediate risk (≥5% and <10%), and high risk (≥10%). In studies evaluating the prognosis of ICU‐admitted pneumonia patients using the CURB‐65 score alone and the CURB‐65 score with the addition of ECOG‐PS, the cutoffs were set at 5% and 20%.^20^ However, since our study includes patients from general wards, we anticipated a lower mortality rate and thus set the cutoffs at 5% and 10%. For the sensitivity analysis, we classified the patients using cutoffs of 5% and 20% and calculated the NRI values.^20^

While calculating the qSOFA score (*n* = 182), we performed multiple imputations using chained equations to account for missing data.^21^ Ten imputed data sets were created and analyzed separately. Subsequently, the results were pooled using Rubin's rules.^22^

We used Stata version 17.0 (Stata Corp, College Station, TX, USA) for all analyses. Statistical significance was set at *p* < 0.05.

## 3 RESULTS

### 3.1 Characteristics of study participants

Of the 1536 enrolled patients, 46 (3.0%) required treatment in the ICU, and 135 (8.8%) died within 30 days. The characteristics of the enrolled patients are summarized in Table 2. The median [IQR] age of enrolled patients was 82.5 (76.0–89.0) years, and 805 (52.4%) patients were women. The mortality rate was higher in patients who required ICU admission than that in those who did not (28.3% vs. 8.2%, *p* = 0.001). The surviving patients were younger and had higher BMIs, a lower prevalence of diabetes mellitus, lower respiratory rates, lower heart rates, and higher systolic blood pressure levels than the deceased patients. Of the total, 975 patients (63.5%) were given a final diagnosis of bacterial infection.

**TABLE 2:** Characteristics of the enrolled patients.

	Death (*n* = 135)	Survival (*n* = 1401)	Data missing
Gender (male/female)	72/63	659/742	0
Age (years), median (IQR)	86 (79–91)	83 (76–89)	0
Consciousness disturbance, *n* (%)	67 (49.6)	496 (35.4)	2
BMI (kg/m^2^), median (IQR)	17.8 (15.6–21.3)	20.5 (18.1–23.1)	5
Body temperature (°C), median (IQR)	36.9 (36.3–37.8)	37.4 (36.7–38.2)	5
Systolic blood pressure (mmHg), median (IQR)	120 (95–144)	128 (109–148)	12
Heart rate (/min), median (IQR)	96 (80–116)	91 (79–105)	5
Respiratory rate (/min), median (IQR)	24 (20–28)	20 (18–24)	171
Diabetes mellitus, *n* (%)	18 (13.3)	361 (25.8)	0
Immunosuppression drugs, *n* (%)	20 (14.8)	126 (9.0)	0
White blood cell count (/μL), median (IQR)	9590 (6360–12,870)	9980 (7040–14,260)	0
C‐reactive protein (mg/dL), median (IQR)	6.9 (2.0–12.7)	6.8 (2.2–13.3)	1
Bacteraemia, *n* (%)	28 (20.7)	242 (17.3)	0

Abbreviations: BMI, body mass index; IQR, interquartile range.

### 3.2 Predictive performance of qSOFA and ECOG‐PS scores

The qSOFA scores were 0 in 385 (25.1%); 1, 633 (41.2%); 2, 276 (18.0%); and, 3, 60 (3.9%) patients. When qSOFA score ≥2 was used as the cutoff point for predicting 30‐day mortality,^1^ the values of Sn, Sp, LR+, LR‐, PPV, and NPV of qSOFA were 44.1% (95% confidence interval [CI]: 35.6–52.9), 76.8% (74.5–79.0), 1.9 (1.5–2.4), 0.7 (0.6–0.8), 15.5% (12.1–19.6), and 93.5% (91.8–94.8), respectively (Table S1A,B). When the qSOFA score was 0, the odds ratio (OR) for predicting 30‐day mortality for qSOFA scores of 1, 2, and 3 were 1.8 (95% CI: 1.0–3.0), 3.3 (1.9–5.7), and 6.9 (3.4–14.2), respectively.

The ECOG‐PS scores were 0 in 351 (22.9%); 1, 273 (17.8%); 2, 245 (16.0%); 3, 375 (24.4%); and, 4, 292 (19.0%) patients. When ECOG‐PS score ≥3 was used as the cutoff point for predicting 30‐day mortality,^20^ the values of Sn, Sp, LR+, LR‐, PPV, and NPV were 65.9% (95% CI: 57.3–73.9), 58.7% (56.1–61.3), 1.6 (1.39–1.83), 0.58 (0.46–0.74), 13.3% (10.9–16.2), and 94.7% (93.0–96.1), respectively. When the ECOG‐PS score was 0, the OR values for predicting 30‐day mortality for ECOG‐PS scores of 1, 2, 3, and 4 were 1.2 (95% CI: 0.6–2.6), 1.7 (0.8–3.4), 2.9 (1.6–5.3), and 4.1 (2.3–7.7), respectively.

### 3.3 Predictive performance of qSOFA and the extended model

**FIGURE 1:**
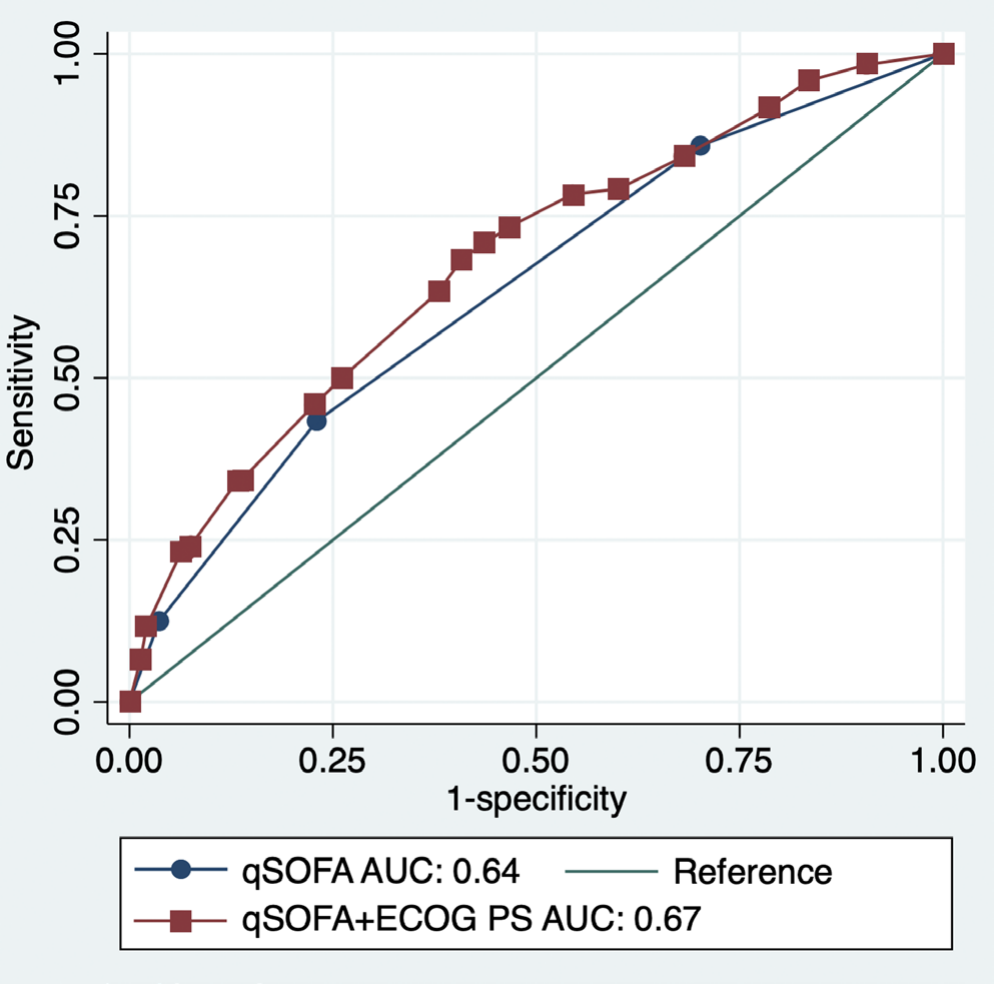
Receiver operating characteristic curves for 30‐day mortality prediction with the qSOFA model and the extended model (qSOFA + ECOG‐PS). Receiver operating characteristic (ROC) curves showing the predictive capacity of the qSOFA model and the extended model (qSOFA + ECOG‐PS) for 30‐day mortality in the older population. AUC, area under the receiver operating characteristic curve; ECOG‐PS, Eastern Cooperative Oncology Group Performance Status; qSOFA, quick Sequential Organ Failure Assessment.

The formulae for the qSOFA and the extended model are listed in Table S2A,B, respectively. The AUC of the extended model was significantly higher than that of the qSOFA model (0.67 [95% CI: 0.62–0.72] vs. 0.64 [0.59–0.69]; *p* = 0.008) (Figure 1).

**FIGURE 2:**
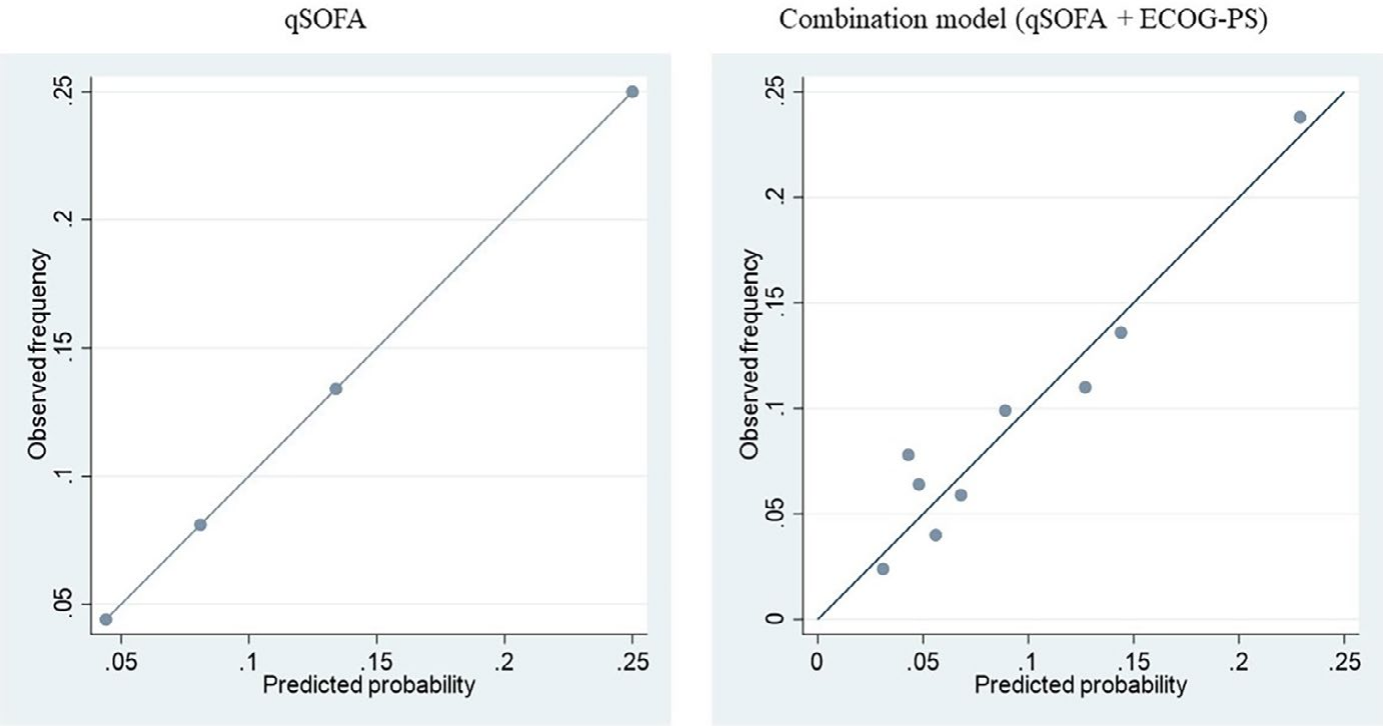
Calibration plots of the qSOFA model and the extended model (qSOFA + ECOG‐PS). Ideally, all groups of predicted probabilities should fit close to the dashed diagonal line (perfect calibration). ECOG‐PS, Eastern Cooperative Oncology Group Performance Status; qSOFA, quick Sequential Organ Failure Assessment.

Calibration plots of the qSOFA and extended model are shown in Figure 2. Calibration of the extended model was evaluated for internal validity using the bootstrap technique, with a slope of 0.91 (95% CI: 0.69–1.14) and an intercept of −0.01 (−0.17–0.17). The extended model showed good calibration, similar to that of the qSOFA model (slope = 0.96 [95% CI: 0.71–1.39], and intercept = −0.01 [−0.18–0.19]), despite its complexity.

The NRI values of the qSOFA and extended models are shown in Table 3. Among the 120 deceased patients, seven initially predicted to have a risk of less than 5% by the qSOFA model were reclassified into the 5%–10% risk category, while 14 patients originally predicted to have a 5%–10% risk were reclassified into the greater than 10% risk category using the extended model. As a result, a total of 21 patients were reclassified into a higher risk category. The categorized NRI value for deceased patients was 0.05 (95% CI: −0.11–0.24), whereas that for those who survived was −0.02 (0.17–0.26), with an overall categorized NRI of 0.03 (−0.06–0.30). The results of the sensitivity analysis conducted using 5% and 20% as the cutoff points were similar to the result of using 5% and 10% (NRI value for event: 0.02 [95% CI: −0.11–0.16], NRI value for nonevent: −0.01 [−0.11–0.29], and overall categorized NRI value: 0.02 [−0.04–0.29], respectively).

**TABLE 3:** Reclassification table comparing the risk of 30‐day mortality predicted by the qSOFA model to that predicted by the extended model (qSOFA + ECOG‐PS).

Survive (*n* = 1234)	qSOFA + ECOG‐PS			Total
	<5%	5%–10%	>10%	
qSOFA				
<5%	261	103	0	364
5%–10%	116	359	111	586
>10%	0	73	211	284
Total	377	535	322	1234

Death (*n* = 120)	qSOFA + ECOG‐PS			Total
	<5%	5%–10%	>10%	
qSOFA				
<5%	10	7	0	17
5%–10%	9	28	14	51
>10%	0	6	46	52
Total	19	41	60	120

Abbreviations: ECOG‐PS, Eastern Cooperative Oncology Group performance status; qSOFA, quick sequential organ failure assessment.

## 4 DISCUSSION

We evaluated the value of adding the ECOG‐PS score to the qSOFA score to predict 30‐day mortality in older patients admitted with suspected infections. Although the qSOFA score had a low Sn for 30‐day mortality, its discriminative performance improved with the addition of the ECOG‐PS score. Furthermore, the NRI analysis showed that the addition of the ECOG‐PS score could improve the predictive performance of the qSOFA score, especially in identifying patients with a poor prognosis.

Vital signs are highly predictive of in‐hospital mortality.^23^ However, previous systematic reviews have shown that the qSOFA score has poor sensitivity (approximately 50%–60%) for predicting mortality in patients with suspected infections.^24^ Therefore, the qSOFA score alone is not recommended as a screening tool in the Surviving Sepsis Campaign: International Guidelines 2021.^25^ In older patients, the Sn of the qSOFA score was low (0.52, 95% CI: 0.16–0.98).^2^ This finding can be attributed to the sudden disruption of homeostasis in life‐threatening conditions, which is potentially associated with the deterioration of homeostatic mechanisms with aging.^26^ In contrast, multimorbidity, comorbidity,^27^ and frailty^28^ are independent risk factors for mortality in patients with infections. Therefore, the addition of these factors may improve the predictive performance of qSOFA. ECOG‐PS is a simple and well‐validated tool for evaluating functional status. The ECOG‐PS scores showed good concordance with those of a complex scale called the Barthel index.^29^ This study aimed to determine the value of adding ECOG‐PS, a functional scale routinely measured in daily practice, to qSOFA in predicting 30‐day mortality in older patients admitted with suspected infections.

Numerous attempts have been made to improve the performance of qSOFA. In patients with sepsis, the addition of the SpO_2_/FiO_2_ ratio to the qSOFA score enhanced the prognostic accuracy of in‐hospital mortality.^30^ Similarly, the inclusion of CRP levels with the qSOFA score improved mortality prediction in adults who presented to the emergency department (ED) with conditions necessitating abdominal surgery.^31^ These studies included both older and younger patients. For patients with characteristics similar to those of the patients included in the present study, incorporation of the biomarker pro‐adrenomedullin into the qSOFA score improved the ability to predict 30‐day mortality.^32^ However, these biomarkers are not widely available in clinical practice. Finally, in a previous study, the predictive performance of the CURB‐65 score for 30‐day mortality was analyzed with and without the addition of the ECOG‐PS score; the Sn of ECOG‐PS score ≥3 with CURB‐65 score ≥3 improved from 15.6 (95% CI: 10.2–22.5) to 30.6 (23.3–38.7).^20^ Our results corroborate the findings of this previous study, although we conducted this study on older patients with suspected infections (not only those with pneumonia).

This study has several clinical implications. Each hour elapsed (from ED admission to antimicrobial administration) was associated with an increase in the odds of inpatient mortality, ranging from 1.01 to 1.16, contingent upon disease severity.^33^ Prognostication is a crucial factor facilitating prompt decision‐making.^3^ To predict prognosis accurately, a composite evaluation that includes the analysis of physical function and comorbidities is necessary. Although the Barthel and Charlson comorbidity indices are well‐known indicators for the assessment of physical function and comorbidity,^34^ they contain multiple items and require calculation. In contrast, the ECOG‐PS score is a simple and quick measure with the same function as the Barthel index.^29^ If applied for early therapeutic intervention, the assessment of patients using ECOG‐PS scores may improve predictive accuracy in daily clinical practice. The NRI values demonstrated the potential of the extended model to classify 5% of the decreased patients into the high‐risk category compared to that of the qSOFA model. Therefore, the extended model demonstrated the potential to identify high‐risk patients who were not identified using qSOFA alone. From these findings, it can be concluded that adding information about physical activity levels, routinely collected in clinical practice when an infection is suspected, to the qSOFA model has the potential to identify an additional 5% of high‐risk patients.

This study had some limitations. First, the inclusion criterion of blood culture examination relies on the decisions made by physicians, which might be considered subjective and could compromise the reproducibility of this study. Additionally, this criterion may include subjects who do not have infections. Nonetheless, this inclusion criterion has been conventionally previously as it is unethical to perform blood culture examinations on all patients.^11^, ^12^, ^13^, ^14^ Moreover, in actual clinical practice, it is often necessary to predict prognosis and develop treatment plans without knowing whether the patient has an infection, making this study's results more reflective of real‐world clinical scenarios. Second, although the ECOG‐PS score is a useful predictor of prognosis, it remains unclear whether intervention strategies such as early initiation of treatment for patients with poor prognosis can effectively improve their clinical outcomes. Third, mortality outcomes were exclusively evaluated by examining medical records and correspondence. Numerous patients continue to attend outpatient clinics postdischarge, facilitating subsequent verification of their survival. Iizuka Hospital and Shirakawa Kosei General Hospital serve as pivotal institutions within the region and receive most notifications on patient deaths. In addition, as 30‐day in‐hospital mortality is predictive of mortality within 30 days of admission, whether during hospitalization or after discharge,^35^ it could be substituted in this outcome setting. Consequently, in cases where patients are discharged within a 30‐day timeframe and lack corresponding outpatient chart records or letters, confirmation of their vital status via telephone is refrained upon. Fourth, counting respiratory rates requires training. In this study, we did not limit the counting of respiratory rates to trained staff. Therefore, the possibility of inaccurate respiratory rate counts exists.

## 5 CONCLUSION

The addition of the ECOG‐PS score improved the performance of the qSOFA score in predicting 30‐day mortality in older patients with suspected infections, particularly in the correct identification of patients with poor prognoses. However, the prognostic value of adding the ECOG‐PS score alone is insufficient. Further studies are required to enhance the prognostic accuracy with a simple score that does not overburden routine practice.

## AUTHOR CONTRIBUTIONS

All authors contributed to the study conception and design. Material preparation, data collection, and analysis were performed by Masataka Kudo, Sho Sasaki, Toshihiko Takada, and Ken‐ei Sada. The first draft of the manuscript was written by Masataka Kudo, and all authors commented on previous versions of the manuscript. All authors read and approved the final manuscript.

## FUNDING INFORMATION

This study was funded by the assistance of the Aso Iizuka Hospital Clinical Research Grant (AIH‐CRG2021‐5).

## CONFLICT OF INTEREST STATEMENT

K.S received a speaker's honorarium from Glaxo Smith Kline K.K. and a research grant from Pfizer Inc., however, these financial interactions are not connected to the present study.

## ETHICS STATEMENT

Ethical approval statement: In compliance with the Ethical Guidelines for Medical and Health Research Involving Human Subjects in Japan, the requirement of informed consent was waived considering the retrospective nature of the study, and the study was approved by the Ethics Committee of Iizuka Hospital (No. 17135).

Patient consent statement: The patients' data underwent anonymization and de‐identification prior to the analysis.

Clinical trial registration: None.

## Supporting information


Table S1.

## Data Availability

The data sets used or analyzed during the current study are available from the corresponding author upon reasonable request.
