# Supplementary material for: Predicting 30‐day mortality in older patients with suspected infections by adding performance status to quick sequential organ failure assessment
Source: J Gen Fam Med. 2025 Feb 5;26(3):238–45. doi: 10.1002/jgf2.764 (PMC12022432; doi:10.1002/jgf2.764)
Supplement: Supplementary file 1 — Table S1. [file JGF2-26-238-s001.docx]

**Supplementary Table 1. Summary of pooled estimates of the ECOG-PS score (A) and the qSOFA score (B) for death**

**(A) ECOG-PS score**^†1^

| Cutoff | Total  (n=1536) | Death  (n=135) | Sensitivity  (95% CI^¶^) | Specificity  (95% CI) | LR+**^‡1^**  (95% CI) | LR-**^‡2^**  (95% CI) | PPV ^§1^  (95% CI) | NPV^§2^  (95% CI) |
| --- | --- | --- | --- | --- | --- | --- | --- | --- |
| ≥1 | 1185 | 120 | 88.9%  (82.3–93.6) | 24.0%  (21.8–26.3) | 1.17  (1.09–1.25) | 0.46  (0.28–0.75) | 10.1%  (8.5–12.0) | 95.7%  (93.0–97.6) |
| ≥2 | 912 | 106 | 78.5%  (70.6–85.1) | 42.5%  (39.9–45.1) | 1.36  (1.24–1.51) | 0.51  (0.36–0.7) | 11.6%  (9.6–13.9) | 95.4%  (93.4–96.9) |
| ≥3 | 667 | 89 | 65.9%  (57.3–73.9) | 58.7%  (56.1–61.3) | 1.6  (1.39–1.83) | 0.58  (0.46–0.74) | 13.3%  (10.9–16.2) | 94.7%  (93–96.1) |
| ≥4 | 292 | 46 | 34.1%  (26.1–42.7) | 82.4%  (80.3–84.4) | 1.94  (1.5–2.52) | 0.8  (0.71–0.9) | 15.8%  (11.8–20.4) | 92.8%  (91.3–94.2) |

**(B) qSOFA score**^†2^

| Cutoff | Total  (n=1536) | Death  (n=135) | Sensitivity  (95% CI) | Specificity  (95% CI) | LR+**^‡1^**  (95% CI) | LR-**^‡2^**  (95% CI) | PPV^§1^  (95% CI) | NPV^§2^  (95% CI) |
| --- | --- | --- | --- | --- | --- | --- | --- | --- |
| ≥1 | 1100 | 115 | 85.2%  (78.1–90.8) | 29.7%  (27.3–32.2) | 1.21  (1.12–1.31) | 0.50  (0.33–0.75) | 10.5%  (8.7–12.4) | 95.5%  (93–97.2) |
| ≥2 | 384 | 60 | 44.1%  (35.6–52.9) | 76.8%  (74.5–79.0) | 1.91  (1.54–2.36) | 0.72  (0.62–0.84) | 15.5%  (12.1–19.6) | 93.5%  (91.8–94.8) |
| ≥3 | 66 | 16 | 12.1%  (7.1–18.8) | 96.5%  (95.4–97.4) | 3.43  (2.01–5.83) | 0.91  (0.86–0.97) | 24.8%  (15.0–37.0) | 92.0%  (90.4–93.3) |

^†1^ ECOG-PS, Eastern Cooperative Oncology Group performance status; ^†2^ qSOFA, quick Sequential Organ Failure Assessment; **^‡1^** LR+, positive likelihood ratio; **^‡2^** LR−, negative likelihood ratio; ^§1^ PPV, positive predictive value; ^§2^ NPV, negative predictive value; ^¶^ 95% CI, 95% confidence interval

**Supplementary Table 2. Prediction of mortality defined using logistic regression analysis.**

**(A)** **qSOFA**

|  | Coefficient | Standard error |
| --- | --- | --- |
| qSOFA |  |  |
| 1 | 0.57 | 0.27 |
| 2 | 1.19 | 0.28 |
| 3 | 1.93 | 0.37 |
| Constant | -3.06 | 0.23 |

**(B)** **qSOFA and ECOG-PS (extended model)**

|  | Coefficient | Standard error |
| --- | --- | --- |
| qSOFA |  |  |
| 1 | 0.41 | 0.27 |
| 2 | 0.95 | 0.29 |
| 3 | 1.59 | 0.38 |
| ECOG-PS |  |  |
| 1 | 0.13 | 0.38 |
| 2 | 0.41 | 0.37 |
| 3 | 0.89 | 0.31 |
| 4 | 1.16 | 0.32 |
| Constant | -3.53 | 0.33 |

qSOFA, quick Sequential Organ Failure Assessment; ECOG-PS, Eastern Cooperative Oncology Group performance status
